# Otolaryngologic manifestations in HIV disease - clinical aspects and treatment

**DOI:** 10.1590/S1808-86942011000300020

**Published:** 2015-10-19

**Authors:** Fernanda Alves Sanjar, Barbara Elvina Ulisses Parente Queiroz, Ivan Dieb Miziara

**Affiliations:** 1 MD. ENT, Fellow in Facial Plastic Surgery - Otorhinolaryngology Department - University of São Paulo; 2 MD. ENT, Fellow in Neurotology - Otorhinolaryngology Department - University of São Paulo; 3 PhD in Otorhinolaryngology - University of São Paulo Medical School; Head of the Stomatology Group - Otorhinolaryngology Department - University of São Paulo

**Keywords:** acquired immunodeficiency syndrome, aids-related opportunistic infections, anti-hiv agents, hiv, oral manifestations

## Abstract

HIV is a pandemic infection with cases notified in almost all countries. The reported prevalence of symptoms in the head and neck is about 80%; otolaryngologists may be the first physician to see such patients. Oral manifestations are the most common, followed by neck and sinus manifestations. Otolaryngologic symptoms may be important signs of antiretroviral therapy failure. Symptoms are present in acute infections and advanced cases.

**Objective:**

To describe new approaches in the past five years for the treatment of the most frequent otolaryngologic manifestations in HIV patients.

**Methods:**

Systematic reviews and case series published in English and Portuguese from January 2002 to October 2007 including the most common otolaryngology manifestations were selected.

**Conclusion:**

Otolaryngologic manifestations are common and ENT specialists must be prepared to identify such cases.

## INTRODUCTION

Retrospective studies suggest that the prevalence of head and neck symptoms is about 80%. Oral manifestations are the most frequent, followed by neck, sinus, and otological findings.^1^ These symptoms may present during acute infections or advanced stages of disease. Oral lesions may be relevant clinical markers of antiretroviral treatment failure.^2^

The purpose of this review of the literature was to describe current advances in the treatment of the main otorhinolaryngological manifestations in HIV-positive patients; the survey comprised papers published from 2002 to 2007.

## MATERIALS AND METHODS

The review was made in Pubmed of the most common published otorhinolaryngological findings. Case series and systematic reviews in English and Portuguese, published from January 2002 to October 2007 were selected.

The main otorhinolaryngological manifestations and their treatment were classified according to disease site and prevalence. *Oral manifestations*: oral candidiasis; pilous leukoplakia; recurrent aphthous stomatitis; neoplasms (Kaposi's sarcoma and lymphomas); conditions caused by herpes simplex, herpes zoster, and the human papillomavirus (HPV); periodontal disease; and oral tuberculosis. *Nasosinusal manifestations*: acute and chronic rhinosinusitis; allergic rhinitis; neoplasms (Kaposi's sarcoma and lymphomas). *Manifestations in the neck*: enlarged lymph nodes; salivary gland involvement. *Otological manifestations*: acute and chronic otitis media; secretory otitis media; external otitis; polyps in the outer ear canal; neoplasm (Kaposi's sarcoma); sensorineural hearing loss; peripheral facial palsy.

## DISCUSSION

### Oral lesions (Table 1)

1 Candidiasis: this is the most common disease in the mouth of HIV/AIDS patients. Its estimated prevalence in Latin America is about 40% of oral lesions (Ranganathan et al.^3^). The causative agent is *Candida albicans*, and this disease is frequently found at the beginning of HIV infection in previously asymptomatic patients; it is also present in 90% AIDS patients. A well established factor for this infection is a decrease in circulating CD4+ T lymphocytes.^2^, ^4^, ^5^, ^6^ Candidiasis is considered as a marker of failure of antiretroviral therapy with or without protease inhibitors.^2^, ^7^ The three most common forms of candidiasis in the mouth of HIV infected patients are the pseudomembranous form, the erythematous form, and angular cheilitis.^5^

**Table 1:** Treatment of the main oral manifestations of the HIV patient

Oral lesions	1^st^ choice of therapy	Other treatment possibilities
	*Angular cheilitis* - nystatin cream 4x/day/14 days.	*Angular cheilitis* - clotrimazole cream 4x/day/14 days.
	*Other forms of candidiasis* -	
Oral candidiasis	CD4>200: nistatina soluçã o 4 - 6ml 4x/dia/7-14 dias.	*Other forms of candidiasis* -
	CD4<200: fluconazole 100 mg (VO) 1x/day/7-14 days. Severe cases: amphothericin B (EV)0.3 mg/kg 1x/day.	CD4>200: itraconazole 200 mg solution 1x/day/7-14 days.
	Topical podophyllin 0.25% - once	
Pillous leukoplakia	Topical retinoids (vitamin A 0.1%) - 2x/day	
	Acyclovir VO 800 mg 6/6h for 20 days	
Herpes zoster	Thalidomide 200 mg/day.	
Periodontal disease	CD4>200: topical acyclovir until lesions disappear.	Acyclovir-resistant patients - foscarnet: continuous infusion for 24 hours (20 mg/kg weight) or intermittent infusion every 8 hours of 60 mg/ml doses in patients with normal kidney function.
	CD4<200/extensive lesions not responding to topical therapy: acyclovir 400 mg (VO) 5x/day. Severe cases: acyclovir EV.	
	Acyclovir 800mg (VO)5x/dia.	Famcyclovir
Herpes zooster	*Cases of viral dissemination* - acyclovir (EV) 10 mg/kg 8/8h.	Valacyclovir
	*Post-herpetic neuralgia* - amitriptyline up to 100 mg/day.	
	Odontologic debridement	
Periodontal disease	Chlorhexidine 0.12% - mouth wash 2x/day	
	for 14 days. *Necrotizing ulcerative periodontitis* - Metronidazole 500 mg 2x/day for 7 to 10 days	

VO: oral route; EV: endovenous route
  Angular cheilitis may occur as erythematous lesions or splits in the corners of the mouth. Erythematous candidiasis presents as atrophic lesions or reddish maculae on the palate and dorsum of the tongue; it may be present in other areas of the tongue. Acute pseudomembranous candidiasis is the most common form; it presents as easily removable white or yellowish plaques on the mucosa, which is hyperemic and may bleed.^5^, ^8^ These two forms may occur simultaneously.^8^ Lesions may extend throughout the mouth in severely immunodeficient patients.^5^ The diagnosis is based on clinical findings.^8^
2 Pilous leukoplakia: this is a rough-surfaced flat whitish lesion on the lateral aspect of the tongue. The cause is the Epstein-Barr virus, and it is generally asymptomatic. It is also a reliable marker of HIV infection and its progression, and of failure of antiretroviral therapy.^2^, ^6^, ^7^ It is also related (like oral candidiasis) with decreased levels of circulating CD4 cells in HIV seropositive patients.^6^, ^9^ It should be noted that this is not an exclusive disease of HIV seropositive patients; it may occur in other immune suppressive states or even in subjects with intact immune systems.^10^
  Therapy aims to reduce the size of this lesion rather than its eradication, and is indicated when pilous leukoplakia interferes with feeding or for esthetic reasons; there are no studies indicating a treatment of choice.^11^
3 Recurrent aphthous stomatitis (RAS): this disease features painful ulcers (aphthae) of varying size and duration located typically on the non-keratinized oral mucosa. The cause is not clear in spite of much research.^12^ There are three main forms: minor, major, and herpetiform. These ulcers may vary by number, size, or duration, although it is not always possible to differentiate them clearly.^12^, ^13^
  Major ulcers are directly associated with the immune status of seropositive patients. Miziara et al., in a study of 94 seropositive AIDS patients with RAS, noted that major ulcers were associated with lower CD4+ e CD8+ counts and increased inversion of the CD4+/CD8+ ratio compared to patients with minor and herpetiform ulcers. Furthermore, the number of patients with major ulcers and absolute CD4+ cell counts below 60 was higher compared to that of patients with herpetiform and minor ulcers.^13^
  Thalidomide is the most effective treatment; the benefit of this drug should be weighed against the risk of adverse effects and teratogenicity.^14^ A multicentric study by Jacobson et al. (2001) compared low intermittent doses of thalidomide (100 mg 3 times a week) with placebo for the prevention of mouth and esophageal ulcers in patients successfully treated with thalidomide in the past, and showed that this drug was not effective for this specific purpose.^11^, ^15^
4 Kaposi's sarcoma (SC): this disease is clinically similar to other vascular lesions such as hemangioma, ecchymosis, or accumulated melanic pigment with spots, plaques or nodules; it may become symptomatic if there is secondary infection or trauma, when it may cause pain and speech and swallowing disorders.^5^ Its incidence has fallen dramatically with the advent of antiretroviral therapy (ART).^16^
  The main sites in the mouth are the palate and the gingival, but it may be found throughout the mouth.^8^ It may be associated with human herpes virus (HPV) 8^8^, ^10^ and 4.^10^ The diagnosis is made with a biopsy; treatment is radiotherapy, local chemotherapy (vinblastine or sodium sulphate tetradecil) and surgery.^5^, ^10^, ^11^ Systemic chemotherapy is an option in advanced cases.^8^
5 Herpes simplex: this disease involves reactivation of a latent virus in the trigeminal ganglion; the main form in seropositive patients is related with a fall in circulating CD4 cells and increased viral load. The typical lesions are vesicles on the lips or mouth that coalesce to form painful irregular ulcers with whitish borders.^5^ The CD4 cell count must be taken into account in the treatment of herpes simplex. If the count is over 200, topical treatment is used for perioral herpes simplex until the lesions clear; if the count is below 200, or if there are extensive lesions, or if the patient did not respond to topical treatment, a systemic antiviral agent such as acyclovir is warranted. Still more severe cases require endovenous medication; intravenous foscarnet may be used in patients with acyclovir-resistant strains.^11^
6 Herpes zoster: the varicella-zoster virus is associated with chronic lesions, frequent relapses, and even death if the virus disseminates in immunosuppressed patients. The trigeminal nerve - particularly its first division - is involved in about 15% to 20% of herpes zoster cases, which leads to ocular and scalp lesions. The mouth and face become involved when the virus reaches the second and third divisions - there may be blisters, vesicles, and unilateral pain. The main sites in the mouth are the palate, the tongue, and the lips.^11^
  Prompt treatment is required because of postherpetic neuralgia, which has a high rate in seropositive patients.^11^ Acyclovir is the drug of choice; it is used intravenously if the virus has disseminated. Famciclovir and valacyclovir are other choices. Post-herpetic neuralgia may be treated with tricyclic antidepressants and intralesional corticosteroids.^11^
7 Lesions caused by the human papillomavirus (HPV): as with oral candidiasis, HPV lesions (papillomas, focal epithelial hyperplasia or condyloma acuminatum) are more frequent when CD4 cell counts are lower;^9^ however, as opposed to oral candidiasis and pilous leukoplakia, HPV infection rates did not decrease following antiretroviral therapy.^5^, ^11^ The most common type is type 32.^17^
  Treatment has not been well defined; acid/caustic agents, cantharidin, topical podophyllin, topical cidofovir, intralesional interferon-α, intralesional bleomycin, topical 5-fluoracil, or surgery (cryosurgery, CO_2_ laser, curettage, or cold blade excision) may be used.^11^
8 Periodontal disease: the main periodontal diseases in HIV/AIDS patients are: linear gingival erythema, necrotizing ulcerative gingivitis, and necrotizing ulcerative periodontitis. Linear gingival erythema presents as a band of intense vascular erythema around the gingival margin, accompanied by discomfort and gingival bleeding. Its frequency is 2 to 6% in developing countries.^3^
  A few studies have suggested a relationship between gingival colonization by *Candida* species and periodontal disease in HIV patients, including linear gingival erythema. The most recent classification of the American Academy of Periodontology has placed linear gingival erythema within the category of gingival disease of fungal origin.^5^, ^18^
  Necrotizing ulcerative periodontitis may be differentiated from necrotizing ulcerative gingivitis by its rapid destruction of soft tissue and bone: teeth may be lost; there may be bleeding, intense pain, and halitosis. The treatment adds antibiotic therapy to the abovementioned measures for linear gingival erythema.^5^, ^11^
9 Oral tuberculosis: this condition is rare; it affects 1.33% of seropositive patients (Miziara et al.^19^)
  There may be single or multiple superficial ulcers or papillomatous lesions usually on the palate and dorsum of the tongue; pain and bleeding may also be present. Oral lesions may be a manifestation of primary or secondary infection.^19^ A biopsy and culture are done for the diagnosis; the treatment is the same in patients with normal or compromised immune status.^19^
10 Lymphomas: the risk of an HIV-positive patient to develop non-Hodgkin's lymphoma is 50 to 200 times that of the general population. Up to 5% of seropositive patients develop this disease if antiretroviral therapy is not started;^20^ only Kaposi's sarcoma is more frequent as an AIDS-associated tumor.
  Seropositive users of injected drugs are especially at risk. The oral manifestations are present in the gingival and alveolar processes, and may extend to the palate. The tonsils may also be involved.
  The Epstein-Barr virus has been found in tissue samples; a biopsy is required for the diagnosis. Therapy consists of combined radiotherapy and chemotherapy.^21^ The most common chemotherapy treatment is the CHOP protocol (cyclophosphamide, doxorubicin, vincristine, and prednisone).^20^

### Nasosinusal manifestations

1 Rhinosinusitis: this disease is very common in seropositive patients - the prevalence ranges from 30 % to 68 %.^22^ Paranasal sinuses are involved because of increased susceptibility to allergic rhinitis, decreased mucociliary clearance, and immune cell dysfunction.^1^, ^23^, ^24^ The microorganisms that cause acute infection in the general population are the same in HIV-infected patients; the treatment, therefore is similar.^1^, ^23^ There is a higher incidence of *Staphylococcus* and *Pseudomonas* in these patients in both acute and chronic infections. Opportunistic bacteria should be taken into account if the disease progresses to immunosuppression, especially if the CD4 T lymphocyte count is below 200 cells.^23^
  The clinical findings of acute infection are similar to those in non-HIV infected patients: headache, fever, purulent nasal discharge, nasal block, and posterior dripping. However, immunosuppressed patients are unable to mount an inflammatory response; thus, the clinical manifestations of sinus disease in these patients may be only chronic coughing, recurring lung infection, fever of unclear origin, or central nervous system infection.^24^ Miziara et al. found purulent rhinorrhea, nasal block, and headache as the most common complaints in patients with chronic rhinosinusitis.^25^
  Imaging methods - computed tomography and magnetic resonance imaging are useful for the diagnosis or if there is complicated sinusitis.
  The medical treatment of acute and chronic cases is similar to that in non-HIV infected patients, consisting of antibiotics against *Staphylococcus* and *Pseudomonas*. The duration of therapy is still controversial; currently, three weeks are recommended for acute cases, and four to six weeks for chronic cases.^1^, ^23^ Topical or systemic decongestants may be useful.^23^
  Antibiotics based on cultures of sinusal content is used in patients that do not respond to therapy, or that present toxemia or with CD4 cell counts below 50 cells/mm^3^ at the beginning of the disease. In non-responding cases, complicated sinus disease, opportunistic infection, or neoplasms should be sought as cases of treatment failure; imaging is useful in such cases.^23^
  Endoscopic surgery may be beneficial in chronic or non-responding cases to reestablish sinus drainage and to support the diagnosis. There are still not enough published data to support surgery in seropositive patients.^23^
2 Allergic rhinitis: allergic conditions, including allergic rhinitis, develop or exacerbate twice as often in seropositive patients.^23^ It is thought to be a consequence of B lymphocyte polyclonal activation, which increases the amount of immunoglobulins, especially Ig E. Increased Ig E levels cause the allergic symptoms in these patients.^1^, ^23^
  The clinical manifestations are similar to those in patients with normal immunity: thick rhinorrhea and nasal block. The treatment may be done with topical corticosteroids and antihistaminic drugs.^1^
  Immunotherapy is a relevant tool for allergic rhinitis patients that do not respond to the usual treatment; its use in seropositive patients, however, remains limited. Randhawa et al. reported a case of a seropositive patient aged 44 years, with non-responding allergic rhinitis, using ART, who was treated with immunotherapy. These authors found that immunotherapy may have caused CD4 T cells to proliferate slightly and the viral load to increase; these findings disappeared with continuation of ART.^26^
3 Neoplasms: the most common neoplasms in seropositive patients are Kaposi's sarcoma (KS) and lymphomas.^24^ KS may affect the mucosa in any part of the upper aerodigestive tract, including the nasopharynx. Lesions may present as plaques or nodules that appear similar to granulation tissue.^24^ Non-Hodgkin's lymphoma may also develop in the nasal cavity, where it may cause nasal block, refractory chronic sinusitis, or other non-specific symptoms such as fever and weight loss.^24^
  Imaging (computed tomography) is useful but cannot differentiate tumors from inflammation. Thus, biopsies are required for a diagnosis if warranted by the clinical examination.^25^

### Manifestations in the neck

1 Enlarged lymph nodes in the neck: this is the most frequent finding in seropositive patients.^1^ The cause varies, ranging from infection to neoplasms; a reactive process is the most common finding.^24^ Prasad et al. found enlarged lymph nodes in the neck in 42% of seropositive patients with otorhinolaryngological manifestations of HIV infection.^1^ Generalized enlargement of neck lymph nodes may be found in over 70% of these patients within the first few months of seroconversion. It may be described as diffuse enlargement of neck lymph nodes involving two or more chains of extra-inguinal lymph nodes for over three months.^1^ Lymph nodes are symmetrical, soft, ranging in size from 1.0 to 5.0 cm, and located mostly in the posterior neck triangle.^1^, ^24^ Although this may be a benign process, fine needle aspiration biopsy may be needed.^1^
  - Presence of constitutional symptoms with no other associated manifestations;
  - Asymmetric or non-generalized lymph node enlargement;
  - Disproportional increase of a single lymph node in patients with generalized lymph node enlargement;
  - Presence of peripheral cytopenia not explained by other factors.
  The incidence of neck involvement by *Mycobacterium tuberculosis* has increased in seropositive patients.^1^ In subjects with advanced disease, 50% to 67% of tuberculous infection will manifest in extra-pulmonary sites, one of the main ones being the neck.^24^, ^27^ Clinical findings may show only an enlarged and hardened neck.^1^ The PPD test and a chest radiograph are needed to confirm or exclude this infection.
  Neoplasms, such as Kaposi's sarcoma or LNH should be investigated if there is rapid increase in the neck volume and hardening of the neck. Histology may be needed in such cases.^1^
2 Involvement of salivary glands: involvement of the parotids has been observed in 5% to 10% of HIV-positive cases.^28^ This may occur because of benign or malign conditions in these patients. Parotid involvement may result in xerostomy, and progressive and painless enlargement of this gland.^24^

Lymphoepithelial cysts are benign tumors that are highly specific for HIV infection; they probably result from secondary gland obstruction due to intra-parotid lymph node enlargement.^24^ These cysts are one of the initial manifestations of this infection, and are rarely found in patients with advanced disease.^30^

Other conditions, such as lymphadenitis, chronic inflammation, or reactive intra-parotid lymph node enlargement, may also cause hypertrophy of the glands.^24^

CT may show these cysts, which are almost always multiloculated. Fine needle aspiration is a safe diagnostic method; although this procedure may reduce the size of cysts, they tend to grow again, which is expected. Surgery is done if there is secondary infection or significant esthetic concerns.^24^, ^29^

In a few cases, CT and/or a physical examination may reveal a solid mass. The risk of malignancy increases markedly in these patients; the Kaposi sarcoma and lymphomas need to be investigated in these cases.^1^ Steel et al. reported a rare case without a prior diagnosis of HIV in which there was chronic bilateral parotid involvement. Pathology of an intra-parotid lymph node revealed Kaposi's sarcoma.^30^

The diffuse infiltrative lymphocytic syndrome is a rare condition caused by infiltration of viscera by CD8 T lymphocytes. Riviera et al. showed by immunohistochemistry that the Epstein-Barr and the HIV viruses are involved in such cases.^31^

It manifests as permanent CD8 T lymphocyte lymphocytosis, neck lymph node enlargement, and unilateral or bilateral parotid edema.^32^ The lungs are often involved, where the clinical presentation is similar to pneumonia due to *Pneumocystis carinii*. Other manifestations are: peripheral neuropathy, lymphocytic infiltration of the liver (hepatitis), myositis, and lymphocytic interstitial nephritis.^33^ A biopsy is essential to define the nature of parotid involvement, 34 and treatment is usually antiretroviral therapy.^35^

### Otologic manifestations

Otologic involvement due to the HIV is rare. However, AIDS because of the HIV includes a variety of clinical conditions that ENT specialists and clinicians should recognize.

Chandrasekhar et al found otologic manifestations in 33% of HIV patients in case series. Of these patients, 34% had fullness of the ear, 32% had dizziness, 29% had hypoacusis, 26% had tinnitus, 23% had otalgia, and 5% had otorrhea. Otitis media was frequent.^37^

Prasad et al monitored 968 HIV patients during 8 years and encountered otologic involvement in 20% of them; chronic otitis media was the most common disease (13%).^1^

Clinical manifestations in the outer and middle ear in AIDS patients vary. They are generally caused by casual otologic infection and opportunistic diseases. The main diseases are: otitis externa, otomicosis, acute otitis media, recurring acute otitis media, secretory otitis media, chronic suppurative otitis media, and herpes zoster oticus. Opportunistic diseases in this context include: malignant otitis externa, chronic otitis media secondary to infection by *Pneumocistis jiroveci*, and neoplasms (Kaposi's sarcoma).^1^, ^36^, ^38^, ^39^

1 Acute otitis externa: its incidence is not higher in AIDS patients compared to the general population.^1^ It is typically caused by *Pseudomonas aeruginosa*, and less frequently by *Aspergillus* and *Proteus*. Excessive skin irritation and trauma are the main predisposing factors. The physical examination shows an edematous and erythematous outer ear canal, which may contain pus, resulting in conductive hypoacusis.^24^ The treatment consists of topical anti-pseudomonas ear drops and frequent cleaning of the outer ear canal.^24^
2 Malignant otitis externa: Chandler described this disease in 1968; it consists of a necrotizing infection of the outer ear canal that may extend to the mastoid and petrous portion of the temporal bone, and cause cranial base osteomyelitis.^36^, ^40^ Although this disease affects mostly elderly and diabetic individuals, immunocompromised patients are also susceptible.^36^, ^41^, ^42^
  *Pseudomonas aeruginosa* is the causative agent in most cases.^41^, ^43^ Reports of *Proteus mirabilis* and *Aspergillus fumigates* in HIV patients have been published.^40^ Ress et al. (1990 and 1995) published a retrospective review of seven AIDS patients diagnosed with malignant otitis externa, of which two had positive cultures of outer ear canal secretions for *Pseudomonas aeruginosa*. The explanations were that patients were immunodeficient, and had previously been given anti-pseudomonas drugs for community-acquired infection.^43^
  *Pseudomonas aeruginosa* infection is opportunistic, and occurs rarely in healthy subjects. This gram-negative bacteria is not normally found in the outer ear canal; it colonizes this area following minor trauma or excessive exposure to water.^41^ In seropositive patients, loss of immunity and the presence of other opportunistic infections (such as CMV), facilitate the development of malignant otitis externa with complications such as cranial base osteomyelitis.^36^, ^40^, ^44^
  The clinical manifestations are similar in seropositive AIDS patients and in healthy subjects.^40^ They consist of intense otalgia that does not respond to analgesics, and that becomes worse at night, temporal or mastoid headaches, and pain in the temporo-mandibular joint. Fever is uncommon. Otorrhea is found in most cases.^40^, ^41^ Granulation tissue in the outer ear canal is generally absent at otoscopy in these patients, which differs from seronegative subjects. The presence of this finding indicates immunity and a favorable prognosis.^41^, ^43^ Cranial nerve involvement may occur at any phase. Other complications are cerebral abscesses, meningitis, venous sinus thrombosis, and others.^40^, ^41^
  The diagnosis is made based on clinical and radiologic findings. Imaging should be done if malignant otitis externa is suspected, preferably computed tomography of the temporal bones. This exam is useful in that it shows the extent of disease, helps define the response to therapy, and may indentify recurrences.^40^, ^43^
  The treatment consists of anti-pseudomonas drugs, such as ciprofloxacin and ceftazidime, for prolonged periods; these drugs considerably reduce the mortality in these patients.^40^, ^41^ If the disease is caused by a fungus, the treatment consists of local cleaning and antifungal agents such as amphothericin B or itraconazol.^41^
3 Otomicosis is a chronic and superficial disease of the outer ear canal; it is generally caused by *Aspergillus niger*.^36^ It may occur following use of antibiotics or during bacterial infection.^45^ The clinical findings are similar to those of bacterial otitis externa, but the diagnosis depends on identifying the fungus in the histopathological exam, as *Aspergillus* is almost always a saprophyte in the outer ear canal.^36^ The treatment consists of cleaning the outer ear canal and topical antifungal agents. If this treatment is ineffective, there may be invasive fungal infection of tissues. In this case, computed tomography or magnetic resonance imaging may be done to assess the extent of the disease.^45^ The treatment of invasive fungal infection consists of surgical debridement and systemic antifungal drugs.^36^, ^45^
4 Acute otitis media and recurring acute otitis media: studies of the temporal bones of seropositive patients have shown varied degrees of inflammation in the middle ear mucosa, even if there are no symptoms, which confirms otologic involvement by the virus.^46^, ^47^
  Acute otitis media is a common infection in seropositive and non-infected children,^48^ and may bring significant morbidity because of hearing loss.^49^ Miziara et al. monitored 459 immunocompromised HIV-infected Brazilian children and reported otitis media as the most common otorhinolaryngological manifestation.^50^ Barnett et al. found that by age 3 years, all HIV positive children will have had at least one episode of acute otitis media. Seronegative children have a 75% prevalence of acute otitis media at the same age.^51^ HIV infected children are more susceptible to acute otitis media than seropositive adults;^52^ Chandrasekhar et al. found that otitis media was present in 23% of these patients.^37^
  Recurring acute otitis media is defined as three or more episodes of acute otitis media within a 6-month period. These episodes are associated with the level of CD4 T lymphocytes, and are more common in immunocompromised hosts.^48^, ^53^
  Several studies have confirmed that the most common microorganisms involved in acute otitis media in seropositive non-immunocompromised children are similar to those in immunocompetent children, namely *Streptococcus pneumonia* and *Haemophilus influenza*.^49^, ^54^ Immunocompromised children have a higher recurrence rate^48^ and Staphylooccus aureus as the possible causative agent.^49^ Acute otitis media secondary to mycobacterial infection - *P. carinii, P. aeruginosa*, and others - has been reported.
  Clinical findings are similar to those in children without the virus. Immunosuppressed individuals have more severe disease and a higher complication rate, such as meningitis and bacteremia.^55^ The treatment given by otorhinolaryngologists is similar to that in immunocompetent patients - antibiotics against common germs. Tympanocentesis is done to collect material for analysis or to increase the coverage against *S. aureus* if therapy fails.^49^
  Zucotti et al have shown that episodes of recurring acute otitis media may be reduced in 100% of children given cefaclor for 6 months.^53^ The heptavalent anti-pneumococcus vaccine was effective and immunogenic in HIV patients, but children with symptomatic virus infection were more susceptible to adverse reactions.^56^
5 Secretory otitis media: otitis media with effusion is related with Eustachian tube dysfunction, which generally is secondary to repeat otitis, atopia, or obstruction by nasopharyngeal factors (usually lymphoid hyperplasia).^24^, ^54^, ^57^ Thus, it is expected for seropositive children with recurring acute otitis media to be more susceptible to persisting middle ear effusion.^58^
  This is an important cause of conductive hearing loss, which may results in language development problems.^54^ Treating the predisposing factors resolves the symptoms in most cases.^57^ Myringotomy and grommet insertion may be necessary.^54^
6 Suppurative chronic otitis media is a chronic inflammation of the middle ear and mastoid, associated with otorrhea and a perforated tympanic membrane. Bernaldez et al. have shown that the prevalence of this condition in seropositive children over a 10-year period was 13.24%, with a 3.31% annual incidence.^59^ The most common microorganisms in this study were *P. aeruginosa* and *Proteus*. Therapy aims at eliminating the germs and improving patient immunity.^59^ Effective antiretroviral therapy reduces the prevalence of chronic otitis media in immunocompromised children, probably by increasing the number of CD4 T lymphocytes.^50^
7 *Pneumocistis jiroveci* infection: *P. jiroveci* is probably the most common pathogen in HIV/AIDS patients.^39^ The main site for this agent is the lung, although extrapulmonary sites have been described, such as the temporal bone.^57^
  Extrapulmonary infection may occur through several routes: hematogenic, lymphatic, contamination in the outer ear canal through the tympanic membrane, or retrograde dissemination through Eustachian's tube. A likely hypothesis for *P. jiroveci* infection in this region is the similar embryological origin of the tympanic membrane and the pulmonary alveoli.^60^
  Clinical manifestations include otalgia and otorrhea; subcutaneous nodules or polyps in the outer ear canal may also be found. There may also be bilateral involvement, otitis media, and mastoiditis.^36^ Lung involvement is not always concomitant.
  This opportunistic infection should be suspected if a seropositive patient presents outer ear canal polyps and does not respond to the usual therapy. In such cases, the lesion should be biopsied to diagnose infection or a malignancy.^61^ The treatment consists of trimethoprim-sulfamethoxazole during three weeks.^36^
8 Kaposi's sarcoma: this is a progressive neoplasm that indicates AIDS.^62^ It may manifest as diffuse lesions in the ear or the tympanic membrane, or as exophytic lesions in the outer ear canal, leading to conductive hearing loss. Therapy ranges from surgical excision to chemo and radiotherapy.^24^
9 Sensorineural hearing loss: its incidence in HIV patients ranges from 23% to 49%. A possible cause is primary HIV infection of the central nervous system or the auditory nerve.^1^ Other causes of sensorineural hearing loss should be sought, such as CNS infection, neoplasms, and ototoxic drugs.^1^, ^24^ Most of these patients present worse sensorineural loss at high frequencies, but with close to normal speech discrimination. Therapy focuses on the specific cause, including hearing rehabilitation and aids.^1^
10 Peripheral facial palsy: the presence of neurological findings is an important cause of morbidity and mortality in seropositive patients. Peripheral neuropathy comprises from 5% to 20% of neurological complications; peripheral facial palsy is rare.^63^, ^64^ This condition may occur at any phase of HIV infection, although it is more common during the initial stages. Patients may present Bell's palsy or the Guillain-Barré syndrome. In more advanced cases with clear immunosuppression, peripheral facial palsy is generally secondary to opportunistic infection of the CNS, such as neurotoxoplasmosis or lymphoma.^23^
